# Peripapillary Vessel Density Reversal after Trabeculectomy in Glaucoma

**DOI:** 10.1155/2018/8909714

**Published:** 2018-06-26

**Authors:** Jung Hee In, So Yeon Lee, Seok Ho Cho, Young Jae Hong

**Affiliations:** Glaucoma Center, Nune Eye Hospital, Seoul, Republic of Korea

## Abstract

**Purpose:**

To evaluate the microvascular changes at the peripapillary area and optic disc in glaucomatous eyes after IOP lowering by trabeculectomy using OCT angiography.

**Methods:**

25 patients with primary open-angle glaucoma (POAG) who underwent trabeculectomy by a single surgeon were evaluated. Using optical coherence tomography angiography, vessel density was evaluated within the whole image, peripapillary, nasal region, and temporal region. Peripapillary vessel density was measured preoperative, 1 week, 1 month, and 3 months postoperatively in POAG patients. Reversal of vessel density was calculated for all analyzed areas.

**Results:**

The intraocular pressure (IOP) decreased from 30.92 ± 6.32 mmHg (range, 18–44) to 12.64 ± 3.35 mmHg (range, 8–22) at 3-month postoperatively. Compared with the preoperative baseline value, whole vessel density, peripapillary vessel density (PvD), and PvD in nasal region and temporal region were significantly increased at 3-month postoperatively. The magnitude of the vessel density reversal was significantly associated with higher preoperative IOP and greater IOP reduction.

**Conclusions:**

A significant increase in the peripapillary vessel density was demonstrated after trabeculectomy using OCT angiography. The reversal of peripapillary vessel density was associated with higher preoperative IOP and greater IOP reduction. Our postoperative results suggest that the ocular perfusion impairment by high intraocular pressure can be improved by IOP reduction, and the reversal of microvasculature may contribute to the rate of glaucoma progression.

## 1. Introduction

Although intraocular pressure- (IOP-) related stress and strain play a central role in glaucoma [1, 2], the potential role of the ocular blood flow in the pathophysiology of glaucoma has been debated and extensively investigated [3, 4].

The association between ocular perfusion and glaucoma has been investigated using several imaging modalities such as fluorescein angiography (FA), Heidelberg retina flowmeter, color Doppler imaging, laser Doppler flowmetry (LDF), and laser speckle flowgraphy (LSFG).

FA has been extensively used to investigate vascular abnormalities in glaucoma, and previous studies reported absolute disc filling defects in glaucoma eyes [5–15].

Optical coherence tomography angiography (OCTA) is a new imaging technique that enables visualization of the retinal and choroidal microvasculatures. It enables visualization of retinal and choroidal blood flow that are not detectable with conventional angiography.

Recent studies using OCTA in which the majority of study population had primary open-angle glaucoma (POAG) demonstrated decreased optic disc and peripapillary perfusion in glaucoma eyes [14, 15]. Structural reversibility after glaucoma treatment is well documented. Reversible optic disc cupping following acute IOP lowering has been known to occur for decades [16–21]. Quigley [16] described an improvement of the optic disc cupping appearance in 40% of children with successful IOP lowering after trabeculectomy.

Also, lamina cribrosa depth can be reduced after surgical IOP lowering in patients with POAG, and the degree of that reduction was associated with the degree of IOP lowering achieved [22].

However, the reversal of the microvasculature in glaucoma has so far not been investigated using OCT angiography.

The present study evaluates the microvascular changes at the peripapillary area in glaucomatous eyes after IOP lowering by trabeculectomy using OCT angiography.

## 2. Methods

### 2.1. Subjects

This prospective study followed the principles of the Declaration of Helsinki and was approved by the Institutional Review Board of the Nune Eye Hospital.

Indications for trabeculectomy were IOP deemed to be associated with a high risk for progression or glaucomatous progression of the visual field or optic disc despite maximally tolerated medications.

Primary open-angle glaucoma (POAG) who were on maximum tolerable medical therapy showed progressive visual loss and judged by glaucoma specialist (YJH) in our center to require trabeculectomy were included.

All participants underwent comprehensive ophthalmic examinations that included best-corrected visual acuity (BCVA), slit-lamp biomicroscopy, gonioscopy, Goldmann applanation tonometry, and dilated stereoscopic examination of the optic disc. They also underwent central corneal thickness measurement using the Pentacam (OCULUS, Wetzlar, Germany), spectral-domain OCT (Cirrus HD-OCT; Carl Zeiss Meditec), standard automated perimetry (Humphrey Field Analyzer II 750; 30-2 Swedish interactive threshold algorithm; Carl-Zeiss Meditec, Dublin, CA), and OCT angiography using the RTVue-XR Avanti scanner (Optovue Inc., Fremont, CA, USA).

For inclusion, all the participants had to meet the following criteria at the initial assessment: best-corrected visual acuity of 20/40 or better, spherical refractive error between −6.0 and +3.0D, cylinder correction within +3D, and a normal anterior chamber and open-angle on slit-lamp and gonioscopic examination.

We defined POAG as the presence of glaucomatous optic nerve damage and associated visual field defects without ocular diseases or conditions that may elevate the IOP. A glaucomatous visual field change was defined as (1) outside normal limit on glaucoma Hemifield test or (2) 3 abnormal points with *P* < 0.05 probability of being normal and 1 point with a *P* < 0.01 by pattern deviation, or (3) pattern standard deviation of *P* < 0.05. A visual field measurement was considered as reliable when false-positive/negative results were <25% and fixation losses were <20%.

Eyes that had undergone previous intraocular surgery or coexisting retinal or neurologic diseases that could affect the visual field were excluded from this study.

All surgeries were performed under pin-point anesthesia. Limbus-based trabeculectomy involving the use of a 2 × 3 mm rectangular half-thickness scleral flap was performed by a single experienced surgeon (YJH) and the same method. All eyes received MMC application intraoperatively. A thin cellulose sponge (approximately 6 × 12 mm) soaked with MMC, concentration of 0.2 to 0.4 mg/cc, was placed over the intended site of the sclera flap for 1-2 min. The concentration and duration were based on the preoperative evaluation of each patient's risk factors for failure (conjunctival hyperaemia, inflammation, and scarring).

The postoperative regimen included topical gatifloxacin 4 times a day for 4 weeks and prednisolone every 2 hours in the first 1 week and then tapered over the next 4 weeks.

All the ocular hypotensive medications were continued up to the time of surgery. The preoperative IOP was defined as the average of 2 measurements within 2 weeks before trabeculectomy. We recorded IOP measurements by Goldmann applanation tonometry at each follow-up visit.

### 2.2. Optical Coherence Tomography Angiography

The OCTA imaging system provides a noninvasive method for visualizing the optic nerve head (ONH) and retinal vasculature. The image acquisition technique is optimized for the split-spectrum amplitude-decorrelation angiography (SSADA) algorithm described in detail by Jia et al. [23].

The system uses an SSADA method to distinguish the movement of red blood cells within the lumen of retinal and choroidal vessels and provide a high-resolution 3-dimensional visualization of retinal microvasculature.

The OCTA characterizes vascular information at each retinal layer as an en face angiogram, a vessel density map, and quantitatively as vessel density (percentage), calculated as the percentage area occupied by flowing blood vessels in the selected region.

The software automatically fits an ellipse to the optic disc margin and calculates the average vessel density within the ONH (referred to as the inside disc vessel density).

The peripapillary region was defined as a 0.75 mm-wide elliptical annulus extending from the optic disc boundary, using the intrinsic software provided by Optovue.

**Figure 1:**
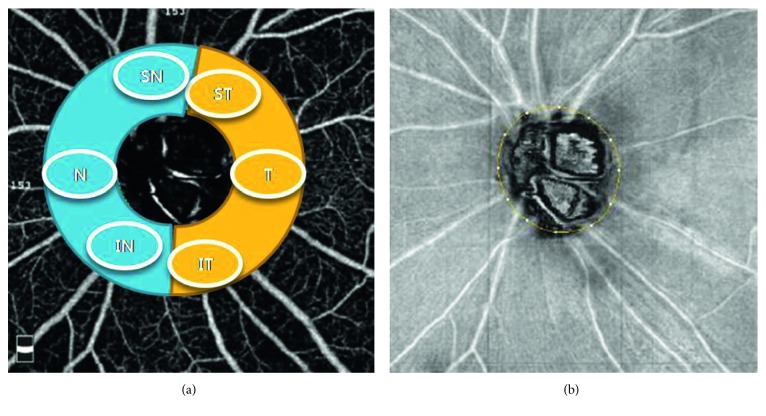
Peripapillary vessel density. Peripapillary vessel density was categorized into superonasal, nasal, inferonasal, superotemporal, temporal, and inferotemporal sectors. Peripapillary vessel density map measurement region defined.

Whole en face image vessel density was measured in the entire 4.5 × 4.5 mm image, and peripapillary vessel density (PvD) was calculated in the region defined as a 0.75 mm-wide elliptical annulus extending from the optic disc boundary (Figure 1).

The peripapillary vessels were analyzed in superficial retinal layers from the radial peripapillary capillary (RPC) segment. The RPC segment extends from the internal limiting membrane to the nerve fiber layer. The peripapillary region was also divided into 6 sectors based on the Garway-Heath map [24] and vessel densities for the entire peripapillary area (average) and each sector were determined.

To compare the changes in the vessel density of the nasal and temporal areas, we defined the average of superotemporal, temporal, and inferotemporal sectors as the temporal region and the average of superonasal, nasal, and inferonasal sectors as the nasal region (Figure 1).

We reviewed scans, and those with poor image quality, as defined by the following criteria, were excluded: (1) a signal strength index <48 (1 = minimum, 100 = maximum), (2) poor clarity, (3) residual motion artifacts visible as an irregular vessel pattern or disc boundary on the en face angiogram, (4) a local weak signal, and (5) RNFL segmentation errors.

The delineation of the disc margin was reviewed for accuracy and adjusted manually, if required.

PvD was examined at 1 day before surgery and 1 week, 1 month, and 3 months postoperatively.

### 2.3. Statistical Analysis

Paired *t*-tests were used to compare preoperative and 3-month postoperative IOP and vessel density.

Logistic regression analysis was used to determine the factors associated with the change of the PvD. Statistical analyses were performed using SPSS 22.0 software (SPSS Inc, Chicago, IL). *P* < 0.05 was considered significant.

## 3. Results

25 patients with POAG who underwent trabeculectomy were included.

The mean age was 61.12 ± 11.85 years (range, 37–82), and 10 subjects were women and 15 subjects were men. The mean refractive error (spherical equivalent) was −1.20 ± 1.70 diopters (range, −6.00 to + 1.50), and the visual field mean deviation was −13.35 ± 6.23 dB (range, −20.82 to −2.28 dB) (Table 1).

The IOP decreased from 30.92 ± 6.32 mmHg (range, 18–44) to 12.64 ± 3.35 mmHg (range, 8–22) at 3-month postoperatively. Compared with the preoperative baseline value, whole vessel density, PvD, and PvD in nasal region and temporal region were significantly increased at 3-month postoperatively (whole image: 36.71 ± 5.81 preoperatively and 38.13 ± 6.21 at 3-month postoperatively (*P*=0.05); PvD: 43.02 ± 6.83 preoperatively and 45.11 ± 6.89 at 3-month postoperatively (*P* < 0.001); PvD in nasal region: 41.59 ± 6.89 preoperatively and 43.43 ± 7.11 at 3-month postoperatively (*p* < 0.008); PvD in temporal region: 43.94 ± 8.13 preoperatively and 45.83 ± 7.60 at 3-month postoperatively (*P* < 0.02)) (Table 2).

**Figure 2:**
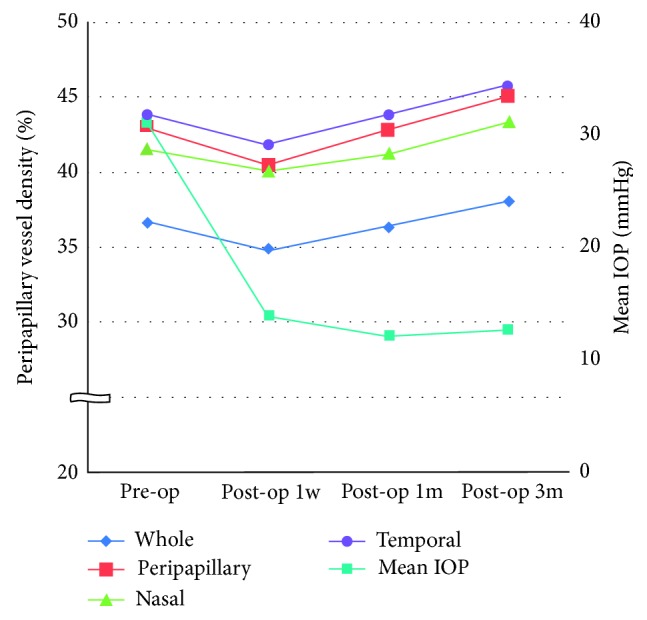
Change in peripapillary vessel density and mean IOP after trabeculectomy.

The PvD decreased slightly at 1-week postoperatively, and thereafter, vessel density increased gradually after postoperative 1 week. At 3 months postoperatively, there was a significant increase compared to preoperative vessel density (Figure 2).

Linear regression showed significant influence of greater IOP reduction on the change of the whole and peripapillary vessel density. And, there were significant influences of higher preoperative IOP and greater IOP reduction on the change of the PvD of nasal and temporal region. In the multivariate analysis, none of the factors were associated with the reversal of PvD (Table 3).

### 3.1. Representative Case

**Figure 3:**
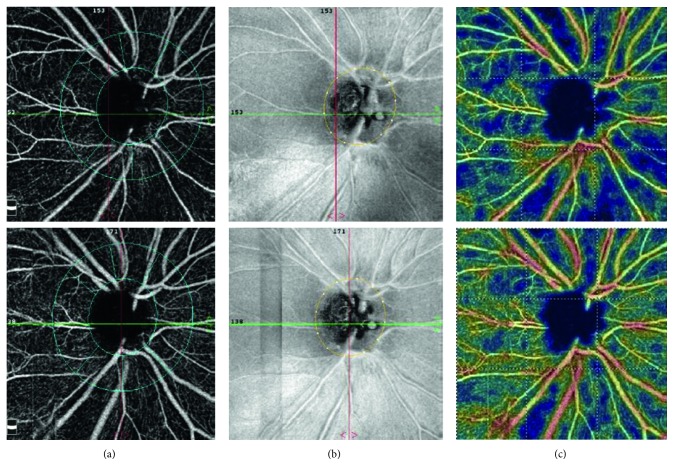
Change in peripapillary vessel density and mean IOP after trabeculectomy. Right eye of a 48-year-old female patient. Top row: preoperative peripapillary vessel density; bottom row: postoperative 3-month peripapillary vessel density. Peripapillary vessel density map of the radial peripapillary capillary layers with the measuring ellipse (a), en face retinal nerve fiber layer image (b), and color-coded peripapillary vessel density map (c). Note the change of the peripapillary vessel density.

Figure 3 shows 1 case that underwent trabeculectomy. Top row image indicated the preoperative PvD and bottom row image indicated the postoperative 3-month PvD. The IOP decreased from 32 to 15 mmHg. The IOP was controlled below 18 mmHg after the surgery. There was significant increase of whole and peripapillary vessel density. Note the reversal of the PvD on color-coded peripapillary vessel density map.

## 4. Discussion

This study investigated the reversal of the PvD after trabeculectomy in glaucomatous eyes.

The results of this study demonstrated that the PvD decreased slightly at 1-week postoperatively and thereafter vessel density increased gradually after postoperative 1 week. At 3 months postoperatively, there was a significant increase compared to preoperative vessel density.

The vessel density reversal was expected in the nasal region where the RNFL defect was not mainly found, but vessel density reversal was similar in the nasal and temporal regions.

A significant reduction of the vessel density in sectors with RNFL defect has been reported [15, 25, 27], but reversal of vessel density has not been associated with RNFL defect location.

As mentioned earlier, the association between ocular perfusion and glaucoma has been investigated using several imaging modalities. Disc blood flow of glaucoma patients was previously investigated by fluorescein angiography, and fluorescein filling defects in the disc have been found in glaucoma patients [5–8].

In previous laser Doppler flowmetry (LDF) and laser speckle flowgraphy (LSFG) studies [9, 13, 26, 28] peripapillary and ONH blood flow decreased in patients with glaucoma compared with controls.

Also, Liu et al. [15] demonstrated higher repeatability and reproducibility of OCT angiography compared with other noninvasive techniques, such as LDF and LSFG. Several studies have suggested that vascular factors can play a pathogenic role in glaucoma, and decreased peripapillary retinal vasculature identified by OCTA is clinically useful to observe the glaucoma progression.

In the present study, the preoperative IOP and the IOP reduction were related to the reversal of PvD postoperatively. This means that the higher preoperative IOP and the greater IOP reduction, the greater the PvD reversal.

Our postoperative results suggest that the ocular perfusion impairment by high intraocular pressure can be improved by IOP reduction and the reversal of microvasculature may contribute to the rate of glaucoma progression.

Recently, Zeboulon et al. [29] measured the influence of IOP lowering by filtering surgery on peripapillary and macular vessel density in glaucoma patients using OCT angiography. They demonstrated a very limited effect of surgically induced IOP reduction on peripapillary and macular vessel density. These results are contradictory to those of this study. The discrepancy between the 2 studies may be attributable to several factors. Firstly, in the study by Zeboulon et al. [29], patients were observed for only up to 1 month after trabeculectomy, so the degree of IOP reduction was maintained for a relatively short period. Secondly, the amount of IOP change differs between the studies. In the study by Zeboulon et al. [29], their patients had lower baseline IOP and lower IOP reduction (baseline IOP: 23.7 mmHg; mean IOP reduction: 11.5 mmHg). In contrast, the baseline IOP was 30.9 mmHg and the mean IOP reduction was 18.3 mmHg at 3 months postoperatively in our study.

And, Holló [30] showed that PvD increased in 6 patients after a medical reduction of IOP of at least 50% from baseline in patients with IOP higher than 35 mmHg.

Furthermore, Alnawaiseh et al. [31] demonstrated the improvement of the flow density of the macular and ONH after cataract surgery with iStent. In the iStent group, the mean IOP was 18.2 ± 3.3 mmHg prior to surgery and 13.2 ± 2.3 at follow-up. Despite the small amount of IOP change, the flow density of the macular and ONH improved after surgery.

Also, Shin et al. [32] evaluated the microvascular improvement after trabeculectomy. In the study, microvascular improvement in OCT angiography was arbitrarily defined as a reduction >30% of the area of vascular dropout on the color-coded vessel density map.

In this study, we evaluated the vessel density reversal on the peripapillary vessel density map.

At 1-week postoperatively, vessel density decreased rather than preoperative vessel density. We speculated that extremely low postoperative IOP after trabeculectomy often cause axial length reduction and change in corneal curvature [33, 34], and this may affect the signal strength of vessel density image in the early postoperative period. Also, postoperative inflammation, postoperative corneal edema, anterior chamber reaction, and hyphema may be variables.

### 4.1. Study Limitation

Our study has several limitations. Firstly, patients were observed for only up to 3 months after trabeculectomy. This was mainly because the IOP commonly re-elevates after this period. It is possible that reversal of the PvD may persist after this period if IOP remained well controlled.

Second, the small number of patients included is explained by the relative difficulty of performing an OCTA scan on the peripapillary area in glaucoma patients.

Third, the limitation inherent to the OCTA technique is that vessel density cannot differentiate large vessels from small capillaries. Thus, if a change in density is observed, it is unclear as to which vascular network is concerned. By observing the color-coded peripapillary vessel density map, nonetheless, we can hypothesize that capillaries are responsible for the vessel density reversal rather than arterioles and venules.

## 5. Conclusion

In conclusion, we demonstrated the reversal of PvD after trabeculectomy in patients with glaucoma.

It seems that reduced ocular perfusion induced by high IOP can be improved by IOP reduction, and the vessel density reversal may contribute to glaucoma progression.

The PvD reversal was affected by preoperative IOP and magnitude of IOP lowering. Further study is required to confirm the influence of the vessel density reversal after IOP reduction on glaucoma prognosis.

## Figures and Tables

**Table 1:** Patients' clinical demographics (*N*=25).

Variables	Mean ± standard deviation
Age (years)	61.12 ± 11.85
Gender (male/female)	15/10
Baseline IOP (mmHg)	30.92 ± 6.32
Spherical equivalent	−1.20 ± 1.70
Central corneal thickness (*µ*m)	558.8 ± 47.23
Average RNFL thickness (*µ*m)	65.4 ± 18.13
Visual field MD (dB)	−13.35 ± 6.23

Values are presented as mean ± SD unless otherwise indicated. *n* = number of eyes; RNFL = retinal nerve fiber layer; IOP = intraocular pressure; MD = mean deviation.

**Table 2:** Pre- and postoperative (3-month) measurements of the IOP and peripapillary vessel density.

		Preoperative (mean ± SD)	Postoperative, 3 months (mean ± SD)	*P* value
IOP (mmHg)		30.92 ± 6.32	12.64 ± 3.35	<0.001
Vessel density (%)	Whole image	36.71 ± 5.81	38.13 ± 6.21	0.05
	Peripapillary	43.02 ± 6.83	45.11 ± 6.89	<0.001
	Nasal sector	41.59 ± 6.89	43.43 ± 7.11	0.008
	Temporal sector	43.94 ± 8.13	45.83 ± 7.60	0.02

**Table 3:** Factors associated with change of peripapillary vessel density.

	Angio vessel density (*P* value)							
	Whole		Peripapillary		Nasal region		Temporal region	
	Univariate	Multivariate	Univariate	Multivariate	Univariate	Multivariate	Univariate	Multivariate
Age (years)	0.187		0.082		0.239		0.124	
Preoperative IOP (mmHg)	0.367		0.197		0.023	0.097	0.036	0.06
IOP reduction	0.028	0.084	0.05	0.178	0.024	0.09	0.05	0.073
Spherical equivalent	0.06		0.069		0.07		0.121	
Central corneal thickness (*µ*m)	0.436		0.236		0.057		0.372	
Average RNFL thickness (*µ*m)	0.227		0.476		0.109		0.162	
Visual field MD (dB)	0.267		0.288		0.284		0.168	
